# From mechanisms to therapies: the multifaceted roles of guanylate-binding protein 2 in immunity, cancer, and beyond

**DOI:** 10.3389/fimmu.2025.1708319

**Published:** 2025-10-16

**Authors:** Wenqi Cui, Tianlu Wang, Juan Feng

**Affiliations:** ^1^ Department of Neurology, Shengjing Hospital of China Medical University, Shenyang, China; ^2^ Department of Neurology, The First Affiliated Hospital of Jinzhou Medical University, Jinzhou, China

**Keywords:** guanylate-binding protein 2 (GBP2), interferon-inducible GTPase, innate immunity, inflammasome, cancer, biomarker, therapeutic target

## Abstract

Guanylate-binding protein 2 (GBP2) is an interferon-inducible GTPase that plays a critical role in innate immunity by defending against viral, bacterial, and parasitic infections through mechanisms such as furin inhibition and inflammasome activation. Beyond infectious disease, GBP2 demonstrates a context-dependent dual role in cancer—acting as either a tumor suppressor or an oncogene by modulating key signaling pathways including JAK-STAT, Wnt/β-catenin, and PI3K/AKT/mTOR. Its dysregulation is also increasingly implicated in autoimmune, neurological, and metabolic disorders, underscoring its promising utility as a diagnostic biomarker and therapeutic target. This review systematically synthesizes current knowledge on GBP2’s structural features, biological functions, and functional duality. We further explore the paradoxical nature of its context-dependent roles and propose a unifying hypothesis to explain its dual functions, while outlining translational strategies to leverage GBP2’s potential in biomarker development and targeted therapies.

## 1 Introduction

Guanylate-binding proteins (GBPs) belong to the dynamin superfamily of GTPases, a group that also includes the very large inducible GTPases, Mx proteins, and other immune-related GTPases (1). In mice, the 11 GBP (GBP1–11) genes are distributed across two chromosomal clusters, whereas humans possess seven GBP genes (GBP1–7) located within a single cluster on chromosome 1. This gene family has ancient evolutionary origins and mediates diverse physiological functions, including immune regulation and host defense against pathogens (2). Among the various GBP family members, GBP2 has gained widespread attention due to its distinctive immunological roles. Structurally, GBP2 has a molecular weight of 65–67 kDa and belongs to the interferon (IFN)-inducible guanylate-binding protein family within the dynamin superfamily of large GTP hydrolases (3). In humans, the GBP2 gene is located on chromosome 1, while in mice, it is found on chromosome 3 (4). Within cells, GBP2 is widely distributed, present in the nucleus, cytoplasm, and perinuclear membrane, where it performs various functions (5). Functionally, GBP2 can be induced by IFN and inflammatory cytokines and is known to influence several signaling pathways, such as phosphatidylinositol 3-kinase (PI3K)/Ak strain transforming (AKT)/mammalian target of rapamycin (mTOR) and wingless-type MMTV integration site family (Wnt)/β-catenin (6, 7). It was initially recognized for its essential role in cell-autonomous immunity against diverse intracellular pathogens, including bacteria, viruses, and parasites (1, 8). Additionally, GBP2 is involved in inflammatory activation during inflammasome assembly and pyroptosis (9, 10). Beyond infectious contexts, dysregulation of GBP2 has been increasingly linked to carcinogenesis. Numerous studies have highlighted GBP2’s dual role in cancer biology, with some defining it as a tumor suppressor across various cancer types (11, 12), while others characterize it as an oncogene (13, 14). We investigate the paradoxical nature of its context-dependent functions and propose a unified hypothesis to elucidate its mechanistic duality. The mechanism of GBP2 involving carcinogenesis includes regulating development and metastasis, immune surveillance and immunotherapy, chemoresistance, immunity against infections, apoptosis, and treatment (9, 15). Moreover, GBP2 contributes to a range of systemic disorders, such as rheumatoid, neurological, and metabolic diseases, as well as hematological conditions and transplant-related pathologies (16, 17). Although earlier research predominantly focused on GBP1, recent investigations have increasingly uncovered unique functions of GBP2, substantially expanding our understanding of its biological and pathological significance. In this review, we provide a comprehensive overview of GBP2, covering its structural features, enzymatic properties, associated signaling pathways, and multifunctional roles, thereby establishing a scientific and theoretical foundation for future research on GBP2.

## 2 GBP2: structures, biological and enzymatic activities and regulation

### 2.1 Structure of GBP2

**Figure 1:**
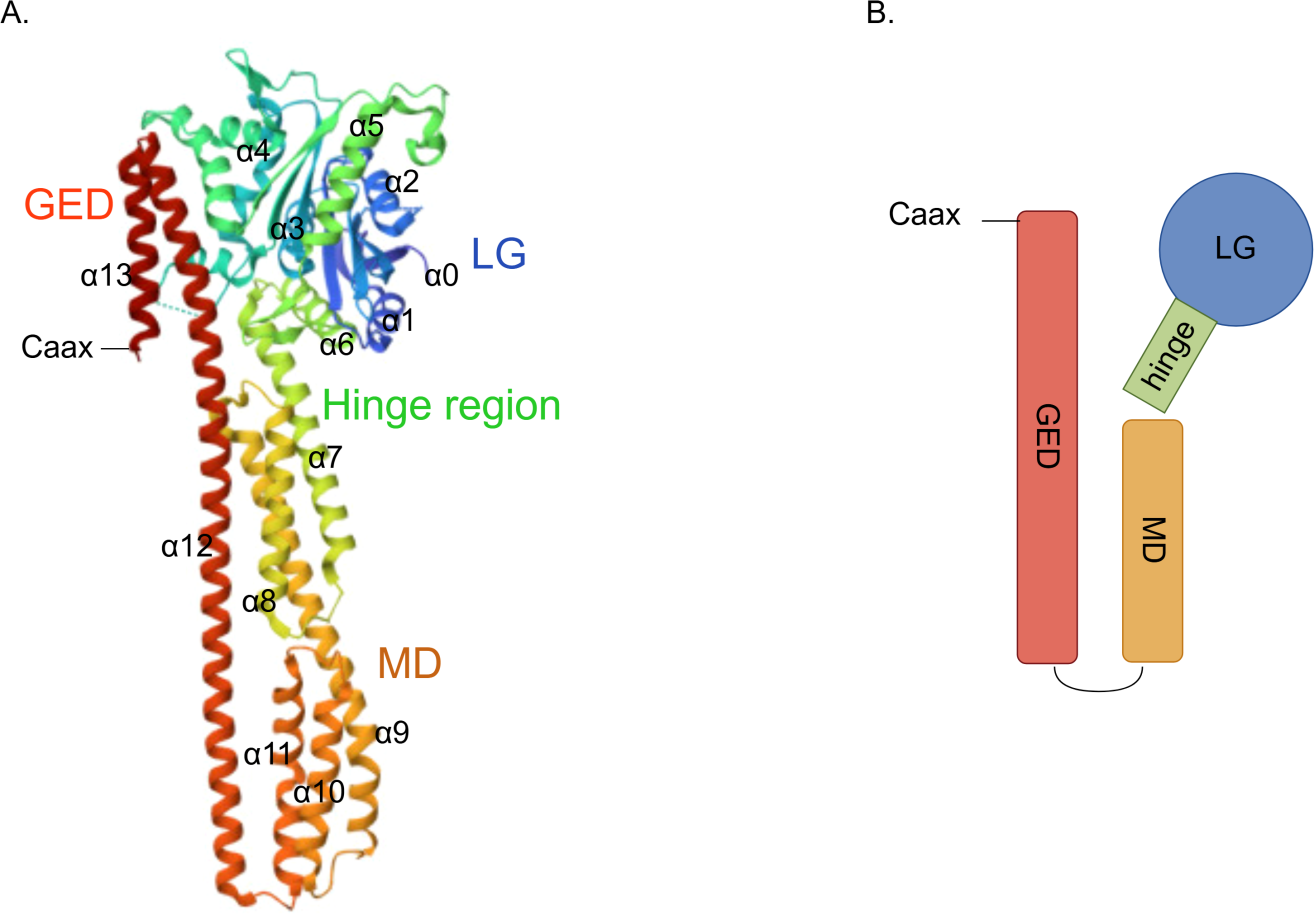
Structure of GBP2. **(A)** Crystal structure depicting the architecture of GBP2 (PDB:7e58); **(B)** A schematic diagram of the GBP2 structure.

GBP2 is a 65 kDa IFN-inducible GTPase that belongs to the dynamin superfamily. Structurally, it comprises three principal domains: an N-terminal globular large GTPase domain (LG) and an elongated purely α-helical region, the latter subdivided into the middle domain (MD) and the GTPase effector domain (GED) (**Figure 1**) (5, 18). The N−terminal LG domain adopts a globular conformation and contains five canonical motifs—G1 (P−loop), G2 (switch I), G3 (switch II), G4 ((N/T)KxD), and G5 (guanine cap)—which together facilitate GTP binding, Mg^2+^ coordination, and hydrolysis (5, 19). Notably, K51A substitutions in the LG domain have been associated with loss of GBP2 GTPase activity (20). A flexible hinge region, formed by α6 and α7 helices and also referred to as the intermediate region, connects the LG domain to the elongated α−helical MD. This hinge is critical for GBP2’s immune functions; mutations of hinge residues, either singly (L307A and P308A) or in combination (L307A/P308A and D306A/L307A/P308A), nearly abolish GBP2’s ability to impair infectivity (21). The MD consists of five α−helices and its interface is essential for furin inhibition, a function important for antiviral activity (21, 22). The GED of GBP2 contains two helices and contacts the LG domain through electrostatic interactions. Importantly, the C−terminus of the GED in GBP2—like that of GBP1 and GBP5—features a CaaX motif that undergoes geranylgeranylation, a key post−translational modification (23, 24). Upon geranylgeranylation, nucleotide binding and hydrolysis regulate the release of the “aaX” tail from the C−terminal α−helical domain. After removal of “aaX”, the carboxyl group at the end of the “C” residue is methylated, enhancing the protein’s capacity to associate with endomembrane organelles (25). GBP2, along with GBP5, is modified by geranylgeranyltransferase I, whereas GBP1 is suggested to be modified by farnesyltransferase (24). Finally, C586S substitutions in the CaaX domain of GBP2 have been shown to prevent its isoprenylation (26).

### 2.2 Biological and enzymatic activities of GBP2

**Figure 2:**
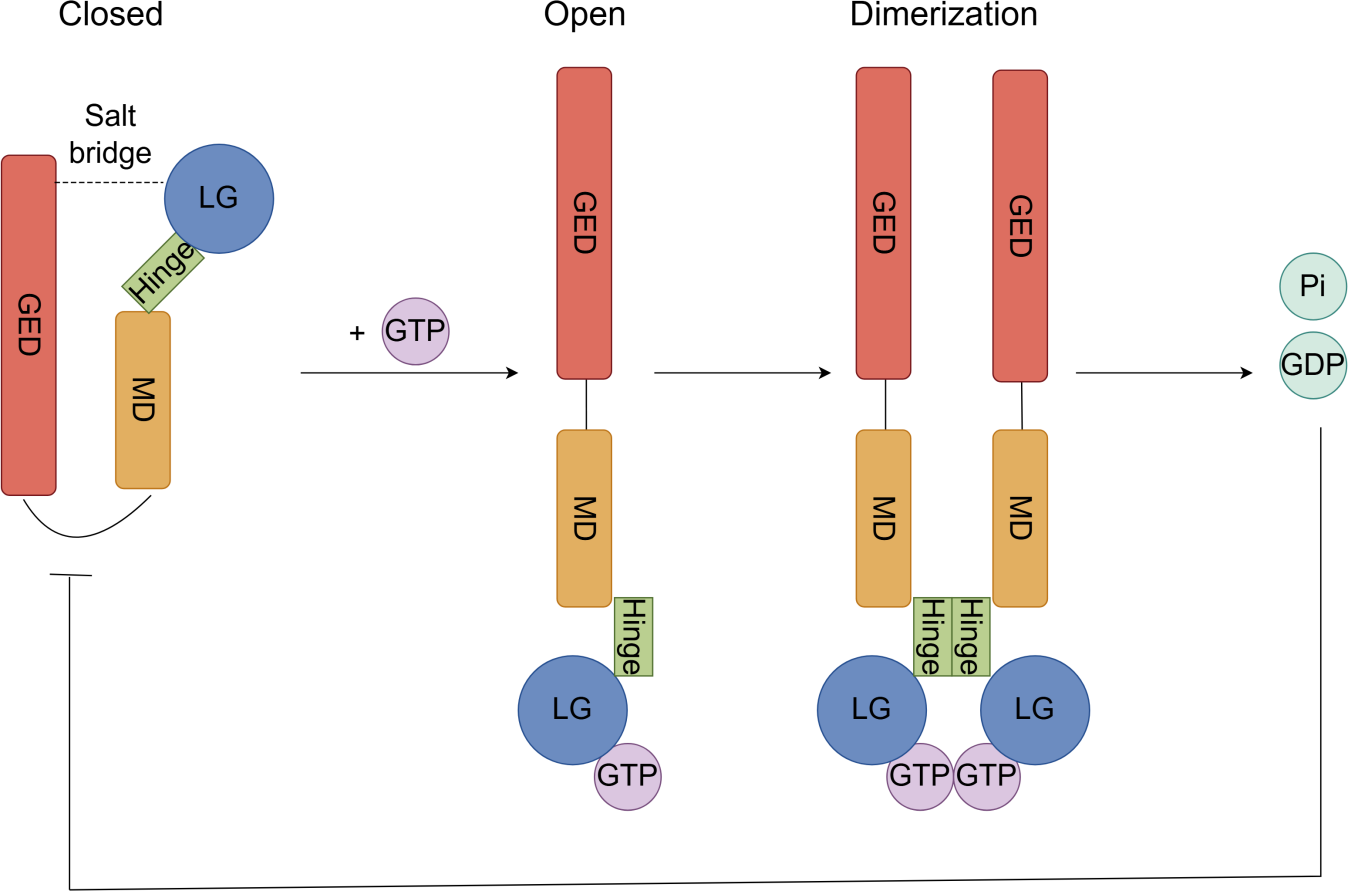
GTP hydrolytic activity and oligomerization process of GBP2. In the closed state, the monomeric structure is stabilized by salt bridges between the LG and GED domains. Upon GTP binding to the LG domain of the open-conformation GBP2, an induced conformational change exposes a hydrophobic helix within the intermediate hinge region, priming the protein for dimerization. Following dimerization, GTP is hydrolyzed to GDP and inorganic phosphate (Pi), and the resulting GDP exerts potent feedback inhibition on GBP2.

GBP2 undergoes conformational changes and/or oligomerization upon guanosine triphosphate (GTP) binding and hydrolysis, processes that are likely crucial for its biological functions (21). Structurally, GBP2 adopts a closed monomeric conformation stabilized by salt bridges within the LG domain and between the LG and GED domains (18). In the presence of substrate, GBP2 dimerizes—a step essential for GTP hydrolysis (27). This dimerization is facilitated by an 18-residue hydrophobic helix within the intermediate region, while tetramerization involves the R−II region (residues 482–556) of the helical domain (28). Notably, heterodimers of GBP2 exhibit extended half-lives compared to homodimers (18). Although GBP2 shares high sequence identity with GBP1, the two proteins display significant functional differences. GBP2 primarily hydrolyzes GTP to guanosine diphosphate (GDP), whereas GBP1 can further process GTP to guanosine monophosphate (GMP). This divergence stems from structural variations in their LG and intermediate domains. The isolated GED of GBP2 hydrolyzes GTP to GDP but remains monomeric; however, when the intermediate region is present, substrate-induced dimerization enables subsequent hydrolysis to GMP. This occurs because, in the free protein, the helix is likely buried within the GTP-binding domain, and substrate binding may expose this helix to promote dimerization (28, 29). Additionally, the two enzymes show distinct feedback inhibition profiles: GDP potently inhibits GBP2 but not GBP1, while GMP strongly inhibits GBP1 but not GBP2 (29). Unlike GBP1, the tetrameric form of GBP2 plays no role in GMP formation (28) (**Figure 2**).

### 2.3 Regulation of GBP2 and related signal pathway

**Figure 3:**
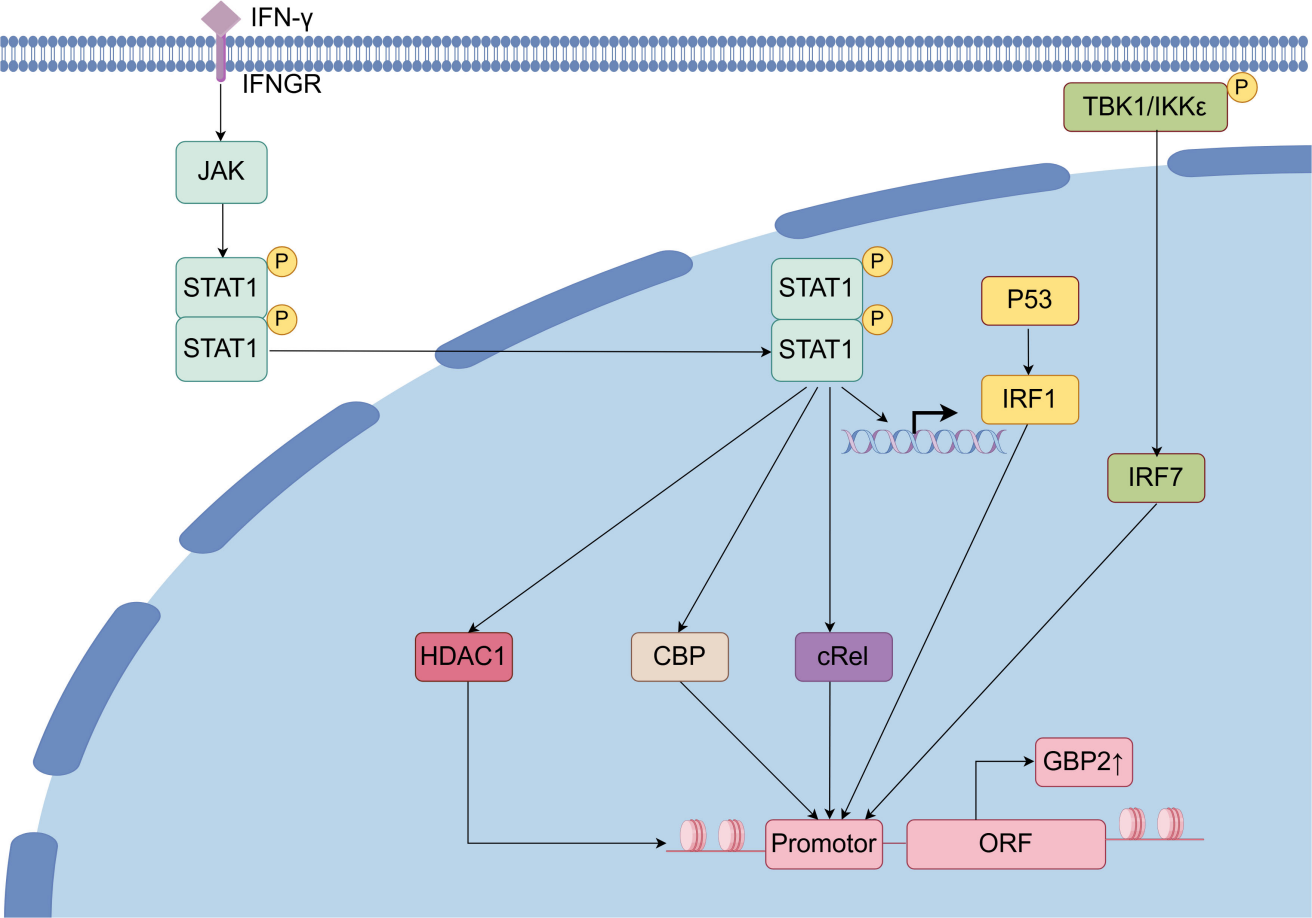
Regulation of GBP2 and related signal pathway. Upon binding of IFN to its receptor, the JAK/STAT signaling pathway is activated, leading to dimerization and nuclear translocation of STAT1. Within the nucleus, STAT1 facilitates the transcriptional activation of GBP2 through the recruitment of co-regulators including histone deacetylase 1 (HDAC1), CREB-binding protein, and cRel, ultimately promoting IRF1-mediated transcription and subsequent translation of GBP2. Additionally, in the presence of TANK-binding kinase 1(TBK1) and/or IKKϵ, IRF7 is activated and further enhances GBP2 expression.

The transcription of the GBP2 gene is dependent on promoter binding sites for Signal transducer and transcription activator 1 (STAT1) dimers and interferon regulatory factor (IRF) family transcription factors (30). STAT1 contributes to GBP2 gene activation through two distinct mechanisms: first, by inducing IRF1 mRNA expression, and second, by directly facilitating GBP2 promoter activation through the recruitment of CREB-binding protein and other histone acetyltransferases, thereby establishing a permissive chromatin environment for RNA polymerase II. Histone deacetylase 1 is also recruited to the GBP2 promoter upon IFN-γ stimulation and is involved in the deacetylation of specific transcriptional activators required for their full activity. Moreover, STAT1 is essential for the association of histone deacetylase 1 with the GBP2 promoter chromatin, which is important for GBP2 expression (31, 32). IRF1, a transcriptional factor regulated by the IFN-STAT signaling pathway, controls the expression levels of GBP2, with its association to the GBP2 promoter occurring after that of STAT1. IRF1 binding takes place independently of STAT1 binding or histone hyperacetylation and may assist in recruiting RNA polymerase II-containing transcriptional complexes (33, 34). Additionally, p53 can upregulate GBP2 expression by stabilizing IRF-1 and promoting the formation of an IRF-1–p53 complex (35). Another IRF family member, IRF7, also regulates GBP2; however, unlike IRF1, its promoter activation depends on the S/T kinase TANK-binding kinase 1 and/or inhibitor of IκB kinase-related IKKϵ (36). Furthermore, the nuclear factor κB (NF-κB) family transcription factor cRel is rapidly recruited to the GBP2 promoter following IFN stimulation and significantly contributes to its transcriptional activation (**Figure 3**) (37).

## 3 The role of GBP2 in defense against bacterial, parasitic, and viral pathogens

**Figure 4:**
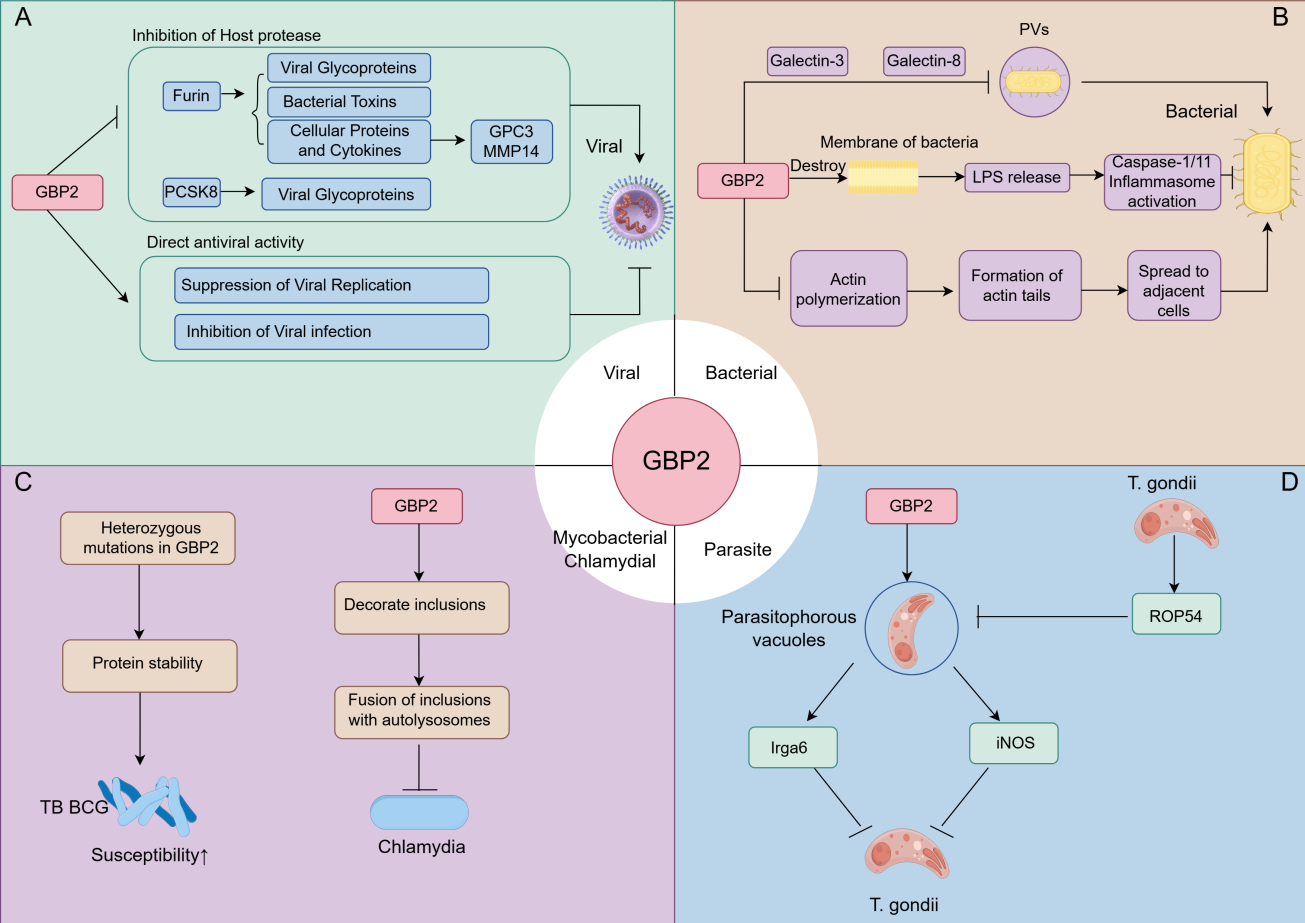
The role of GBP2 in defense against bacterial, parasitic, and viral pathogens. **(A)** During antiviral responses, GBP2 targets viral infectivity by inhibiting host proteases furin and PCSK8, and directly restricts bacterial replication and infectivity; **(B)** In antibacterial immunity, GBP2 is recruited to pathogen-containing vacuoles (PVs) via Galectin-3/8 to exert antimicrobial effects. It also disrupts bacterial membrane integrity, releasing LPS to activate inflammasomes, and inhibits actin polymerization to suppress bacterial dissemination; **(C)** GBP2 modulates susceptibility to M. tuberculosis Bacille Calmette–Guérin (BCG) and restricts chlamydial infection by regulating inclusion bodies; **(D)** In response to Toxoplasma gondii, GBP2 controls recruitment to parasitophorous vacuoles, promotes activation of Irga6 and inducible nitric oxide synthase(iNOS), and contributes to antiparasitic defense. Conversely, T. gondii secretes the effector rhoptry effector protein 54 (ROP54) to counteract GBP2 recruitment to parasitophorous vacuoles.

GBP2 plays a major role in cell-autonomous innate immunity against bacterial, parasitic, and viral infections (5). The mechanisms of GBP2 in defensing against bacterial, parasitic, and viral pathogens were summarized in **Figure 4**.

### 3.1 The role of GBP2 in anti-viral immunity

GBP2 exerts broad antiviral activity by targeting multiple stages of the viral life cycle. It suppresses the replication of diverse viruses—including HIV, avian influenza A, murine leukemia virus, Zika virus, measles virus, and Marburg virus—by inhibiting the host protease furin and attenuating its proteolytic activity, thereby impeding the cleavage and maturation of viral envelope glycoproteins (1, 2, 38). Notably, the inhibition of furin by GBP2 may have implications beyond viral infection, as several bacterial toxins, such as anthrax toxin protective antigen and diphtheria toxin, also depend on furin-mediated activation (39). Furin itself is involved in multiple cellular processes, including the proteolytic activation of cytokines, collagens, hormones, and growth factors; GBP2 can also inhibit furin-mediated cleavage of substrates such as glypican-3, a proteoglycan that regulates cell growth and proliferation, and matrix metalloproteinase-14. Beyond furin, GBP2 reduces the infectivity of viral particles bearing the PCSK8-dependent glycoprotein of Lassa virus (38). The N-terminal GTPase activity of GBP2 is critical for its antiviral function. For instance, GBP2 suppresses ectromelia virus replication in a dose-dependent manner, an effect abolished by the GTP-binding-deficient mutant K51A (25). Similarly, in murine macrophages, GBP2 mediates IFN-γ–triggered anti-murine norovirus activity, whereas the R48A and K51A mutants attenuate this effect, indicating a potential requirement for GTPase activity. Murine norovirus nonstructural protein 7, however, can co-localize with GBP2 in the cytoplasm and antagonize its anti-viral function (20). GBP2 also contributes to host defense against other viruses. It interferes with SARS-CoV-2 spike cleavage and significantly inhibits infection by early-lineage strains such as Wuhan-Hu-1 and VIC (40). In addition, GBP2 inhibits the replication of vesicular stomatitis virus and encephalomyocarditis virus (41). Notably, GBP2 expression is associated with dengue disease severity. During dengue infection, endothelial cells upregulate GBP2 as an initial protective response, which mitigates viral impact via reduced oxidative stress. However, in severe cases, GBP2 levels decline during the defervescence phase, likely due to heightened oxidative stress, and this decrease correlates with increased plasma leakage—a hallmark of severe dengue. Thus, GBP2 plasma levels, alongside clinical symptoms, may serve as a biomarker for dengue severity (42). Furthermore, GBP2 expression can be modulated by viral elements integrated into the host genome. For example, HIV-1 infection activates a cryptic transcription start site within the long terminal repeat 12C repeat upstream of the GBP2 gene, leading to a unique transcript variant and enhancing cytokine-responsive expression of this antiviral gene (43).

### 3.2 The role of GBP2 in antibacterial immunity

GBP2 plays a significant role in cell-autonomous immunity against bacterial infections. Many invasive bacteria establish pathogen-containing vacuoles as intracellular niches for replication, and GBP2 contributes to immunity by facilitating pathogen-containing vacuol recognition and mediating the transport of host defense proteins to these compartments. The disintegration of such vacuoles attracts Galectin-3, -8, and -9, though to date the functional consequences of this recruitment have only been characterized for Galectin-8 (44). GBP2 identifies bacterial secretion systems as “patterns of pathogenesis” associated with pathogen-containing vacuols, and its delivery varies depending on the bacterial species and galectin involved. For instance, GBP2 delivery to *Legionella*-containing vacuoles requires the bacterial Dot/Icm system, whereas its delivery to *Yersinia* vacuoles depends on hypersecretion of translocon proteins. Galectin-3 assists GBP2 localization to pathogen-containing vacuoles by forming a complex with it, and Galectin-8 also promotes this process, albeit less efficiently (45). The C-terminal CAAX motif of GBP2 enhances its recruitment to *Francisella novicida* compared to GBP1, likely owing to GBP2’s longer lipid anchor, which increases membrane stability in bacteria containing long-chain lipid A (46). GBP2 also targets cytosolic *F. novicida* and promotes bacteriolysis, indicating that beyond defending against vacuolar pathogens, GBPs facilitate ligand presentation by directly attacking cytosolic bacteria (47). Additionally, infection with Gram-negative bacteria such as *Salmonella*, *Citrobacter*, *Chlamydia*, and *Escherichia* in innate immune cells activates the caspase-11 inflammasome, a process that primarily requires GBP2 (48). GBP2 has been shown to promote caspase-11 and caspase-1 inflammasome activation in response to Gram-negative bacteria and intracellular lipopolysaccharide (LPS) (49). In infection with *Moraxella catarrhalis*, GBP2 acts as the dominant GBP driving inflammasome activation. It is recruited to cytosolic bacteria, exhibits dose-dependent bactericidal activity, and disrupts bacterial membranes to release LPS, thereby facilitating NOD-like receptor protein 3 (NLRP3) inflammasome activation (50). Similarly, GBP2 contributes to *Brucella abortus* DNA-mediated inflammasome activation, although full host protection depends on cooperation among multiple GBPs (51, 52). Beyond direct bactericidal effects, GBP2 restricts bacterial dissemination by modulating actin dynamics. For example, the cytosolic bacterium *Burkholderia thailandensis* exploits host actin to induce cell fusion, spreading to form multinucleated giant cells that support its replication. Accordingly, GBP2 deficiency led to significantly increased susceptibility to *B. thailandensis* in both macrophages and mice. Recruitment of GBP2 to bacteria limited the actin tail formation required for bacterial motility and cell fusion. Its association with non-motile bacteria suggests that GBP2 directly—or via actin regulators—restricts bacteria-mediated actin polymerization. Mechanistically, this inhibition required both GTPase activity and the CAAX membrane localization domain (49). *Pseudomonas aeruginosa* frequently causes chronic airway infections in cystic fibrosis patients, most isolates of which have defective type III secretion systems (T3SS). GBP2 can detect T3SS-mutant *Pseudomonas* and contribute to bacterial killing by activating caspase-11 and regulating noncanonical NLRP3 inflammasome activation and IL-1β release (53). Notably, some pathogens have evolved countermeasures against GBP2: the *Shigella flexneri* effector IpaH9.8 induces ubiquitination and proteasomal degradation of GBP2, thereby disrupting GBP-mediated immunity (54). Moreover, bacteriophages—natural predators of bacteria—have been used to treat bacterial infections. In the murine macrophage cell line RAW 264.7, Bacteriophage vB_SauM_JS25 significantly upregulated GBP2, suggesting that phage infection can induce GBP2 expression, potentially enhancing host innate immunity to promote bacterial and viral clearance (55).

### 3.3 GBP2 in mycobacterial and chlamydial infections

In tuberculosis, GBP2 is consistently downregulated and has been identified as a hub gene with diagnostic potential. Its expression is significantly reduced in tuberculosis patients, showing promise for treatment monitoring (56–58). Conversely, in pleural tuberculosis, GBP2 expression is elevated in pleural fluid and demonstrates over 80% accuracy in discriminating tuberculosis from other causes of pleural effusion (59). Genetic studies have linked compound heterozygous mutations in GBP2 to increased susceptibility to *Mycobacterium bovis* Bacille Calmette–Guérin infection, as these mutations affect both protein stability and mRNA splicing (60, 61). Additionally, GBP2 may influence host responses to *Mycobacterium leprae*. Reversal reaction—a major cause of tissue injury and disability in leprosy—results from rapid cell-mediated immune responses against *M. leprae*. The upregulation of GBP2 mRNA in both the peripheral blood transcriptome and lesion transcriptome of reversal reaction patients suggests that GBP2, along with other GBP family members, contributes to the host antimicrobial response against mycobacteria (62).

GBP2 also confers resistance to *Chlamydia trachomatis* in IFN-γ–stimulated human macrophages by promoting the fusion of chlamydial inclusions with autolysosomes. Knockdown of GBP2 abrogates IFN-γ–mediated inhibition of bacterial growth (63). Notably, GBP2 efficiently decorates *C. trachomatis* inclusions, but is absent from *C. muridarum* inclusions in both murine embryonic fibroblasts and macrophages at various time points post-infection (64). Moreover, GBP2 was found to be upregulated when reinfection was compared to primary infection for Chlamydia pneumoniae (65).

### 3.4 The role of GBP2 in antiparasitic immunity

GBP2 functions as a crucial immune effector molecule that confers resistance against parasites. It has been shown to interact with both the parasitophorous vacuole membrane and the parasite itself following vacuole permeabilization (8). A common human intracellular pathogen in this context is *Toxoplasma gondii* (*T. gondii*), with approximately 30% of people harboring asymptomatic persistent infection (66). GBP2 plays a specific and non-redundant role in controlling *T. gondii*, and its deficiency in mice increases susceptibility to infection. The loss of GBP2 alone is sufficient to confer such susceptibility, underscoring its essential function (67). GBP2 modulates recruitment to the parasitophorous vacuoles of *T. gondii* and contributes to parasite growth restriction (66). It localizes specifically to these vacuoles, thereby hindering parasite replication and dissemination. The C-terminal domain of GBP2 is necessary and sufficient for its vacuolar recruitment (67). Additionally, GTP binding, multimerization, and GTPase activity are critical for efficient recruitment; both the binding mutant D182N and the GTPase-defective mutant K51A exhibit nearly abolished localization (68). The function of GBP2 further involves coordinating other immune effectors. It positively regulates the recruitment of Irga6 to the parasitophorous vacuoles of *T. gondii*, although this process can be inhibited by direct and specific interactions of RabGDIα with GBP2 via the lipid-binding pocket (69). Furthermore, GBP2-positive parasitophorous vacuoles are enriched with inducible nitric oxide synthase, which is essential for controlling parasite burden (8). GBP2 also co−localizes with GBP1 in *T. gondii*−infected cells, suggesting that the two proteins may act together at the parasitophorous vacuoles (70). Notably, *T. gondii* has evolved countermeasures: the rhoptry effector protein 54 promotes infection by modulating GBP2 loading onto parasitophorous vacuoles (71). Conversely, evidence also points to species−specific roles for GBP2, as it was reported to be dispensable for IFN−gamma−induced toxoplasmosis resistance in human foreskin fibroblasts (72). The functions and potential mechanisms across various pathogens are summarized in **Table 1**.

**Table 1:** Mechanisms of GBP2-mediated host defense against pathogens.

Pathogen name	Mechanism	References
Viruses		
HIV, Avian influenza A, Murine leukemia, Zika, Measles, Marburg virus	Inhibits the virus-dependency factor furin, impeding the cleavage and maturation of envelope glycoproteins.	(1, 2, 38)
Lassa virus	Reduces the infectivity of viral particles bearing the PCSK8-dependent glycoprotein.	(38)
Ectromelia virus	Suppresses replication in a dose-dependent manner requiring GTPase activity (abrogated by K51A mutant).	(25)
Murine norovirus	Mediates IFN-triggered antiviral activity, requiring GTPase activity (attenuated by R48A/K51A mutants). Antagonized by viral NS7 protein.	(20)
SARS-CoV-2 (Wuhan-Hu-1, VIC)	Interferes with viral spike protein cleavage.	(40)
Vesicular stomatitis virus, Encephalomyocarditis virus	Inhibits viral replication.	(41)
Dengue virus	Associated with an initial protective response (reducing oxidative stress).	(42)
Bacteria		
Legionella pneumophila	Delivery to pathogen-containing vacuoles is dependent on the bacterial Dot/Icm secretion system.	(45)
Yersinia species	Delivery to pathogen-containing vacuoles requires hypersecretion of Yersinia translocon proteins.	(45)
Francisella novicida	Recruitment enhanced by the C-terminal CAAX motif. Targets cytosolic bacteria to promote bacteriolysis.	(46, 47)
Gram-negative bacteria (e.g., Salmonella, Citrobacter, Escherichia, Chlamydia)	Promotes caspase-11 and caspase-1 inflammasome activation in response to intracellular bacteria and LPS.	(48, 49)
Moraxella catarrhalis	Recruited to cytosolic bacteria, exhibits dose-dependent bactericidal activity and disrupts bacterial membranes to release LPS, facilitating caspase-11–NLRP3 inflammasome activation.	(50)
Brucella abortus	Contributes to DNA-mediated inflammasome activation.	(51, 52)
Burkholderia thailandensis	Recruited to bacteria to restrict the formation of actin tails, limiting intracellular motility and cell-to-cell spread. Requires GTPase activity and CAAX domain.	(49)
Pseudomonas aeruginosa (T3SS mutant)	Detects and contributes to killing of bacteria with defective Type 3 Secretion Systems (T3SS).	(53)
Shigella flexneri	Pathogen countermeasure: The bacterial effector IpaH9.8 induces ubiquitination and proteasomal degradation of GBP2.	(54)
Mycobacteria		
Mycobacterium tuberculosis	Unclear (expression is downregulated during infection).	(56–58)
Mycobacterium bovis Bacille Calmette–Guérin	Unclear (genetic mutations in GBP2 linked to increased susceptibility).	(60–62)
Mycobacterium leprae	Unclear (upregulated during reversal reaction, suggesting a role in host response).	(62)
Chlamydia		
Chlamydia trachomatis	Promotes the fusion of chlamydial inclusions with autolysosomes in IFN-γ–stimulated macrophages.	(63)
Chlamydia muridarum	Unclear.	(64)
Chlamydia pneumoniae	Unclear.	(65)
Parasites		
Toxoplasma gondii	Recruits to the parasitophorous vacuole (PV). Hinders parasite replication. Coordinates recruitment of other immune effectors	(8, 66–69)

## 4 GBP2 in inflammation and immune regulation

Innate immunity is the first line of host defense against infection. Beyond its direct antimicrobial roles against viral, bacterial, mycobacterial, and parasitic pathogens, GBP2 also exerts important regulatory functions within the innate immune system.

### 4.1 Regulation of immune cell function and polarization

**Figure 5:**
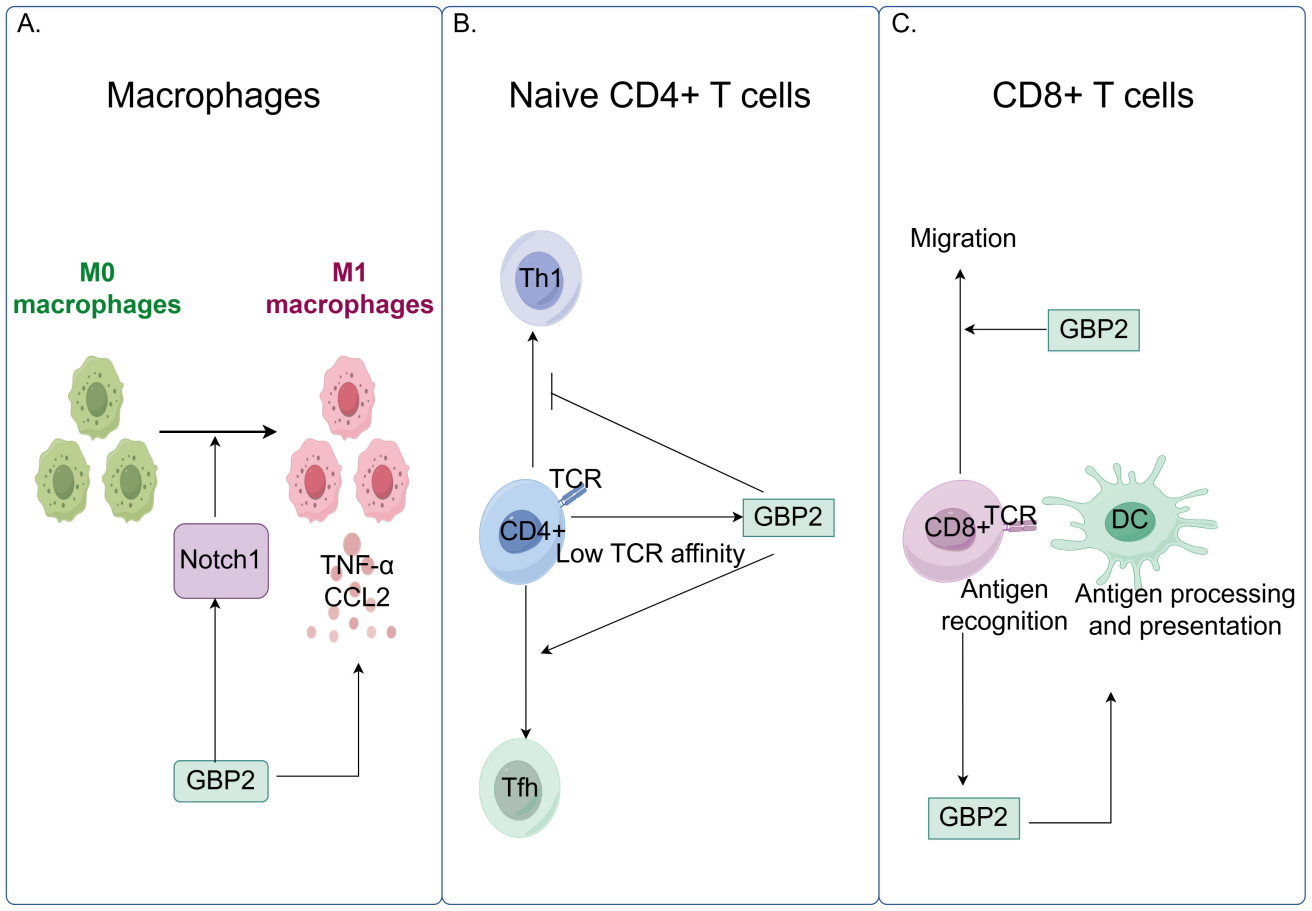
GBP2 modulates innate and adaptive immune responses by regulating macrophage polarization and T cell function. **(A)** GBP2 promotes macrophage polarization toward the pro-inflammatory M1 phenotype through activation of the Notch1 signaling pathway and stimulates the production of pro-inflammatory cytokines, including TNF-α and C-C motif chemokine ligand 2 (CCL2); **(B)** In naïve CD4^+^ T cells, low T cell receptor (TCR) affinity and signaling induce GBP2 expression, which promotes T follicular helper (Tfh) cell differentiation while suppressing T helper 1 (Th1) differentiation; **(C)** GBP2 enhances CD8^+^ T cell migration. Antigen recognition triggers GBP2 upregulation, which further facilitates IFN-γ-induced antigen presentation.

GBP2 influences the activation and phenotypic polarization of multiple immune cell types. In macrophages, silencing GBP2 prevents polarization into pro-inflammatory M1 phenotypes and reduces the production of pro-inflammatory cytokines such as tumor necrosis factor-α (TNF-α) and C-C motif chemokine ligand 2, while showing no effect on M2 macrophage markers or anti-inflammatory cytokines. Moreover, GBP2 promotes M1 macrophage polarization through activation of the neurogenic locus notch homolog protein 1 (Notch1) signaling pathway (73). In T lymphocytes, GBP2 modulates differentiation and function. When naïve CD4^+^ T cells encounter microbial peptide:MHCII complexes on dendritic cells, their differentiation into various T helper subsets is influenced by T cell receptor affinity. GBP2 is induced as a T cell receptor -responsive protein: under low T cell receptor affinity and signaling, it promotes T follicular helper differentiation by restraining aerobic glycolysis. Alternatively, GBP2 may repress Th1 differentiation, with high T cell receptor affinity enabling T cells to overcome this inhibition (74). In CD8^+^ T-cell responses, antigen recognition in epithelial target cells upregulates IFN-regulated genes including GBP2 (75). Furthermore, in murine microsatellite-stable colorectal cancer models, deletion of GBP2 impaired CD8^+^ T-cell migration and reduced IFN-γ-induced antigen presentation and C-X-C motif chemokine ligand10/11 expression (**Figure 5**) (76). GBP2 also regulates inflammatory signaling pathways. It suppresses TNF-α-induced expression of matrix metalloproteinase-9 by inhibiting NF-κB transcriptional activity and Rac1 activation. Mechanistically, GBP2 interferes with p65 binding to κB consensus sites and the metalloproteinase-9 promoter, and dampens Rac activation—a pathway that, when constitutively active, can restore NF-κB signaling even in the presence of GBP2 (77).

### 4.2 GBP2 in inflammasome activation and pyroptosis

**Figure 6:**
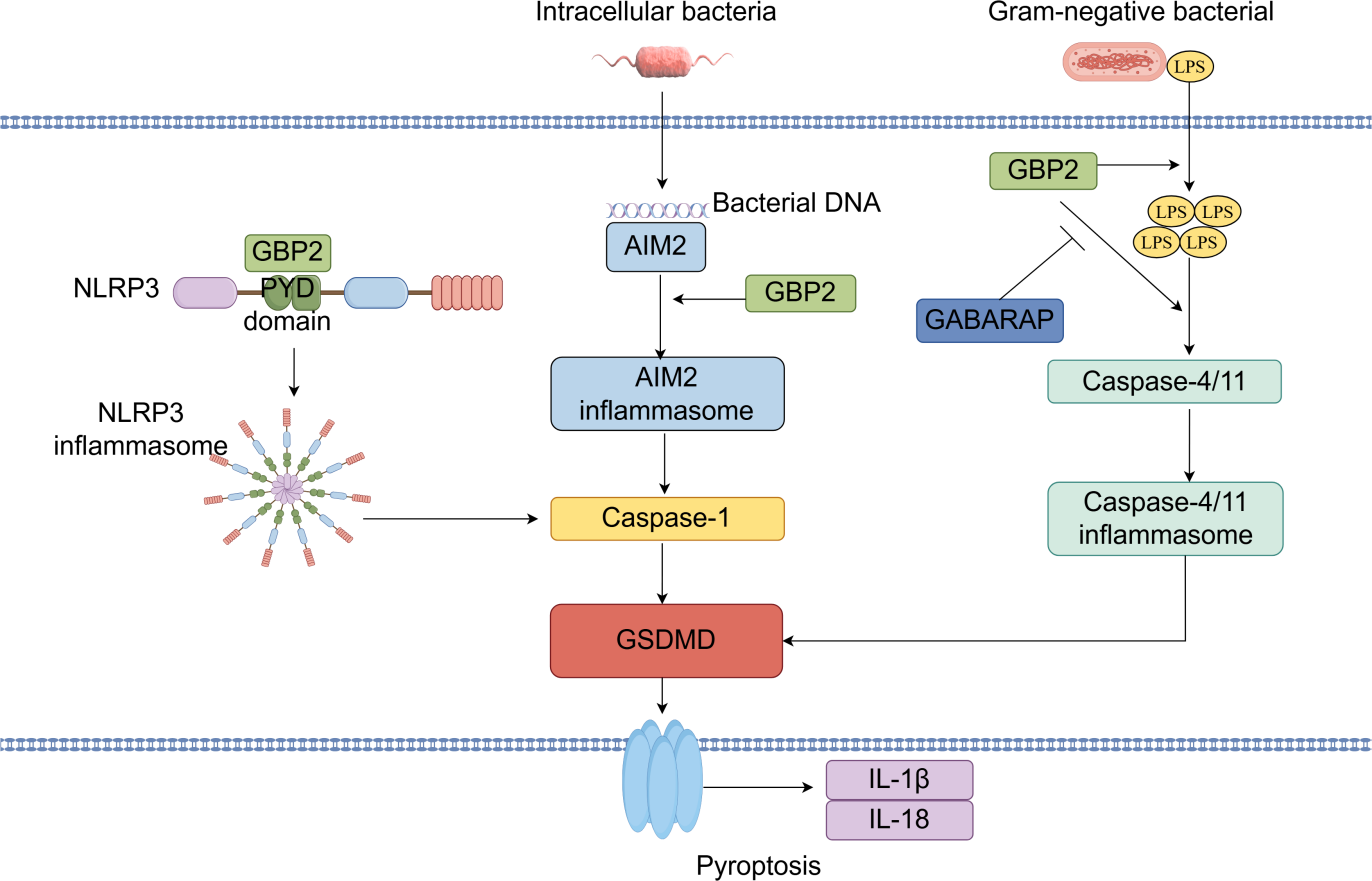
GBP2 in inflammasome activation and pyroptosis: A Central Coordinator and Effector. GBP2 facilitates the extraction of lipopolysaccharide (LPS) from bacterial outer membrane vesicles, promoting caspase-4/11 recruitment and activation via LPS binding and aggregation. This caspase-11 inflammasome activation is negatively regulated by GABARAP-family autophagy proteins. Moreover, GBP2 is essential for AIM2 inflammasome activation upon cytosolic bacterial infection. Additionally, GBP2 enhances NLRP3 inflammasome activation through direct binding to its N-terminal PYD domain.

The inflammasome is an intracellular signaling complex that, upon recognition of pathogens or physiological abnormalities, drives caspase-1 activation, pyroptosis, and the release of pro-inflammatory cytokines IL-1β and IL-18 (10). Pyroptosis is a lytic and inflammatory form of programmed cell death triggered by cytosolic sensing of pathogens or danger signals. It is commonly initiated when intracellular LPS activates human caspase-4 or mouse caspase-11, leading to gasdermin-D cleavage and pore formation (78). GBP2 contributes to this process by facilitating inflammasome assembly and activation. It promotes the release of LPS from bacterial outer membrane vesicles, enabling LPS to interact with caspase-11 (79). Studies indicate that GBP2, along with other guanylate-binding proteins, coordinates caspase-4 recruitment and activation: GBP1 initiates platform assembly, GBP2 and GBP4 facilitate caspase-4 recruitment, and GBP3 promotes its activation (80). Additionally, GBP2 enhances caspase-4 activation by binding LPS and promoting its aggregation (81). GBP2-dependent caspase-11 inflammasome activation has been suggested as essential for preventing LPS-mediated and polymicrobial septic shock *in vivo*. Direct cytosolic sensing of LPS by caspase-11 triggers inflammasome activation, which can lead to lethal sepsis in mice; thus, inhibiting this pathway is critical for preventing septic shock. The regulation of caspase-11 activation by GBP2 is itself modulated by GABA type A receptor-associated protein autophagy proteins, which negatively regulate GBP2-dependent inflammasome activation to protect against sepsis. Depletion of the GABA type A receptor-associated protein subfamily in macrophages enhances IL-1β production and pyroptosis in response to LPS transfection, outer membrane vesicle treatment, or Gram-negative bacterial infection (48). Notably, although GBP2 deficiency significantly reduces pyroptosis, IL-1β/IL-18 secretion, and caspase release in macrophages, its loss does not impair caspase-11 activation as severely as deficiency of the entire GBP cluster, indicating partial functional redundancy among GBP family members (15, 82). Beyond its role in caspase-4/11 activation, GBP2 also modulates absent in melanoma 2 (AIM2) inflammasome signaling (9). The AIM2 inflammasome, which is critical for host defense against cytosolic DNA viruses and bacteria, recognizes double-stranded DNA and induces caspase-1-dependent pyroptosis along with IL-1β and IL-18 release (47). GBP2 promotes *Francisella novicida*-mediated AIM2 inflammasome activation but is dispensable for AIM2 activation triggered by transfected DNA (47). Furthermore, GBP2 can promote NLRP3 inflammasome activation in an isoprenylation-dependent manner, potentially through direct interaction with the PYD domain of NLRP3 (26). Although bacterial outer membrane vesicles carrying flagellin can activate the NOD-like receptor family, CARD domain-containing 4 inflammasome, GBP2 is not required for NOD-like receptor family, CARD domain-containing 4 activation induced by *Salmonella typhimurium*-derived vesicles (79). Finally, recent evidence shows that GBP2 can drive pyroptosis independently of GBP1, underscoring its non-redundant functions in specific contexts (**Figure 6**) (81).

## 5 GBP2 in cancer

GBP2 is expressed at variable levels across diverse human malignancies and exhibits context-dependent roles in tumor progression. Its expression correlates with clinical outcomes in a cancer-type-specific manner. Notably, low GBP2 expression is associated with poor prognosis and increased metastasis in colorectal cancer, while elevated GBP2 levels are linked to worse survival in patients with glioma, glioblastoma, clear cell renal cell carcinoma, pancreatic adenocarcinoma, bladder cancer, and cutaneous melanoma (6, 13, 14, 83–87). Due to its strong prognostic value, GBP2 has been incorporated into multi-gene prognostic models for several cancers, including bladder urothelial carcinoma, breast cancer, pancreatic carcinoma, ovarian cancer, primary central nervous system lymphoma, and cutaneous melanoma (88–95). These models demonstrate significant efficacy in predicting patient overall survival.

### 5.1 Dual roles of GBP2 in tumor development, metastasis, and invasion

GBP2 plays context-dependent roles in tumor progression, with evidence supporting both promotive and inhibitory functions across different cancer types. In glioblastoma multiforme, GBP2 overexpression significantly promotes cell migration and invasion *in vitro*, whereas its silencing produces the opposite effect. This pro-invasive activity is mediated through fibronectin, which is markedly induced by GBP2 at both mRNA and protein levels. Inhibition of the STAT3 pathway prevents GBP2-promoted fibronectin induction and cell invasion (13). Similarly, in clear cell renal cell carcinoma, GBP2 overexpression enhances phosphorylation of STAT2 and STAT3, triggering janus kinase(JAK)-STAT signaling and promoting cell migration and invasion (96). In glioma, GBP2 knockdown impairs proliferation and migration. Mechanistically, GBP2 directly interacts with kinesin family member 22 (KIF22) and regulates glioma progression through KIF22/epidermal growth factor receptor signaling *in vitro* and *in vivo (* 97). Furthermore, GBP2 expression shows significant association with the mitogen-activated protein kinase and Wnt signaling pathways, both known to promote tumor occurrence and malignancy in various cancers (98). GBP2 also influences fibroblast proliferation dynamics. In NIH3T3 fibroblasts, GBP2 expression induces faster growth rates, with the highest-expressing clones showing approximately 50% reduction in doubling time. These GBP2-expressing fibroblasts exhibit increased growth rate, partial loss of contact inhibition, and enhanced ability to grow in reduced serum conditions—effects potentially associated with its regulation of Rho family GTPases. Notably, an GBP2 mutant (S52N) with reduced GTP-binding capacity fails to produce these phenotypes when expressed at levels comparable to wild-type protein (99). Additionally, GBP2 is associated with the proliferative and neoplastic phenotype of esophageal squamous cells. Although not a conventional transcriptional target of p53, GBP2 may influence cellular capacity to maintain sustained proliferation and survival (35).

Conversely, several studies link GBP2 to reduced metastatic potential. In osteosarcoma, GBP2 downregulation enhances migration and invasion (100), while low GBP2 expression correlates with poor prognosis and metastasis. GBP2 also modulates STAT family signaling through phosphorylation events, promoting STAT1 phosphorylation by competing with SH2-containing protein tyrosine phosphatase 1 for STAT1 binding (76). In skin cutaneous melanoma, GBP2 dampens development and metastasis by inhibiting Wnt/β-catenin signaling, suggesting its utility as both a prognostic biomarker and anti-metastatic target (6). Besides, dynamin-related protein 1-dependent mitochondrial fission has a key role in breast cancer cell invasion (101). In breast cancer, GBP2 blocks dynamin-related protein 1 translocation from the cytosol to mitochondria, thereby attenuating dynamin-related protein 1-dependent mitochondrial fission and cancer cell invasion (12). GBP2 further suppresses migration and invadosome formation by modulating Rho GTPase activity. In mouse mammary carcinoma 67NR cells, GBP2 promotes cellular projections and filopodia formation, indicating cell division cycle 42 activation, while also upregulating RhoA activity. GBP2 knockdown results in a rounded cell morphology with lamellipodia, consistent with Rac1 activation (102). Additionally, GBP2 hinders AKT activation during cell spreading on fibronectin and suppresses Rac activation essential for this process. Both IFN-γ and GBP2 inhibit platelet-derived growth factor-initiated cell spreading, accompanied by suppressed Rac activation (103). The full-length GBP2 requires GTP binding and potentially dimerization to effectively inhibit cell spreading, but these properties alone are insufficient—isoprenylation is also essential. While GBP2 typically undergoes modification with the C20 geranylgeranyl isoprenoid, addition of the C15 farnesyl moiety also inhibits cell spreading (104). Moreover, GBP2 forms a complex with PI3K p110 subunit, which is crucial for inhibiting cell spreading, as PI3K activation during spreading is curtailed by GBP2 (103).

### 5.2 The roles of GBP2 in apoptosis of tumor

Dysregulated apoptosis is a hallmark of cancer development. The B cell lymphoma gene 2 (BCL-2) protein family acts as a pivotal regulator of apoptosis, maintaining the delicate balance between cell survival and death. This family includes both antiapoptotic and proapoptotic members. Upon receiving death signals, proapoptotic proteins such as BCL-2 antagonist/killer 1 (BAK), BCL-2 associated X, and/or BCL-2-related ovarian killer undergo oligomerization, leading to mitochondrial outer membrane permeabilization (105). This process results in the release of apoptotic molecules including cytochrome c, which subsequently triggers caspase activation. GBP2 contributes to tumor regulation by modulating apoptotic pathways. It interacts with myeloid cell leukemia 1—a key antiapoptotic protein in the BCL-2 family—via its BH3 domain. This interaction competitively inhibits myeloid cell leukemia 1’s pro-survival function in chronic myeloid leukemia cells, thereby liberating BAK from myeloid cell leukemia 1 binding. In addition, GBP2 significantly upregulates BAK expression by suppressing the PI3K/AKT pathway (4). Correspondingly, knockdown of GBP2 was shown to significantly increase proliferation and reduce apoptosis in acute myeloid leukemia cells. Furthermore, miR-221 promotes acute myeloid leukemia cell proliferation partly through targeting GBP2 and regulating PI3K/AKT pathway activation (106). Pyroptosis, a form of inflammatory programmed cell death, also plays a role in cancer cell death and the tumor immune microenvironment through host-tumor crosstalk. As previously discussed, GBP2 is involved in the regulation of pyroptosis (107), highlighting its multifaceted role in cell death mechanisms relevant to cancer.

### 5.3 The roles of GBP2 in immune environment of tumor

GBP2 has been associated with immune surveillance, immunotherapy response, immune regulation, and defense against viral infections in the tumor microenvironment. Immune checkpoint therapy represents a predominant strategy for many advanced cancers, though its efficacy depends on the presence of sufficient T cells. By blocking the programmed cell death 1 (PD-1)/programmed cell death ligand 1 (PD-L1) axis to hinder immune evasion, CD8^+^ T cells can effectively drive tumor eradication (108). Currently, the role of GBP2 in immunotherapy remains controversial. One perspective supports its beneficial role, proposing GBP2 as a combination target with checkpoint blockade due to its influence on anti-PD-1 response, regulation of PD-L1 expression, and effects on CD8^+^ T cells. Increasing GBP2 expression enhances anti-PD-1 response and inhibits colorectal cancer growth (76). It was identified as a hub gene linked to CD8^+^ T cells in an immune-related gene score predicting lymphoma subtypes and treatment response (109), and correlates with T cell-related genes in breast cancer defense (110). Conversely, other evidence suggests GBP2 may be detrimental to immunotherapy outcomes. Studies indicate that GBP2 significantly correlates with increased expression of multiple immune checkpoints (including PD-1 and PD-L1) and with CD8^+^ T cell distribution in the renal cell carcinoma tumor microenvironment, potentially promoting cancer progression (14, 111). A signature combining GBP2 predicts metastasis and immune infiltration in prostate cancer (112). Moreover, in clear cell renal cell carcinoma, high GBP2 expression is associated with greater infiltration of CD3^+^, CD8^+^, and CD68^+^ immune cells, along with elevated expression of immune checkpoint markers PD-1 and cytotoxic T lymphocyte antigen 4, as validated by Opal multiplex immunohistochemistry (96). In gastric cancer, elevated GBP2 expression is linked to poor prognosis, immune modulators, infiltrating immune cells, biomarkers, and immunotherapy response (83, 113). However, one study in esophageal cancer found no association between inflammatory infiltrate and GBP2 expression (35). Regarding immune surveillance, most characteristic immune checkpoints are significantly more expressed in high-GBP2 groups compared to low-GBP2 groups (84). GBP2 may also influence cancer progression through immune regulation against viral infections. Given that many malignancies—such as liver cancer with HBV, nasopharyngeal cancer with EBV, and cervical cancer with HPV—are associated with viral infections, and considering that various viruses induce IFN production upon host invasion, GBP2 likely plays a role in host defense. Whether the pathology of cancers with high GBP2 expression involves viral infection merits further investigation (84). Gastric cancer, one of the most common and lethal cancers worldwide, is primarily caused by Helicobacter pylori infection. GBP2 was among the highly upregulated genes in H. felis-infected mice, potentially promoting cancer progression by enhancing angiogenesis, proliferation, migration, metastasis, invasion, and tumorigenicity (114). The paradoxical regulatory effects of GBP2 in the immune microenvironment may be explained by the fact that high GBP2 expression could represent both immune activation and a compensatory immunosuppressive state, such as through upregulation of checkpoint molecules.

### 5.4 The roles of GBP2 in therapy of tumor

**Figure 7:**
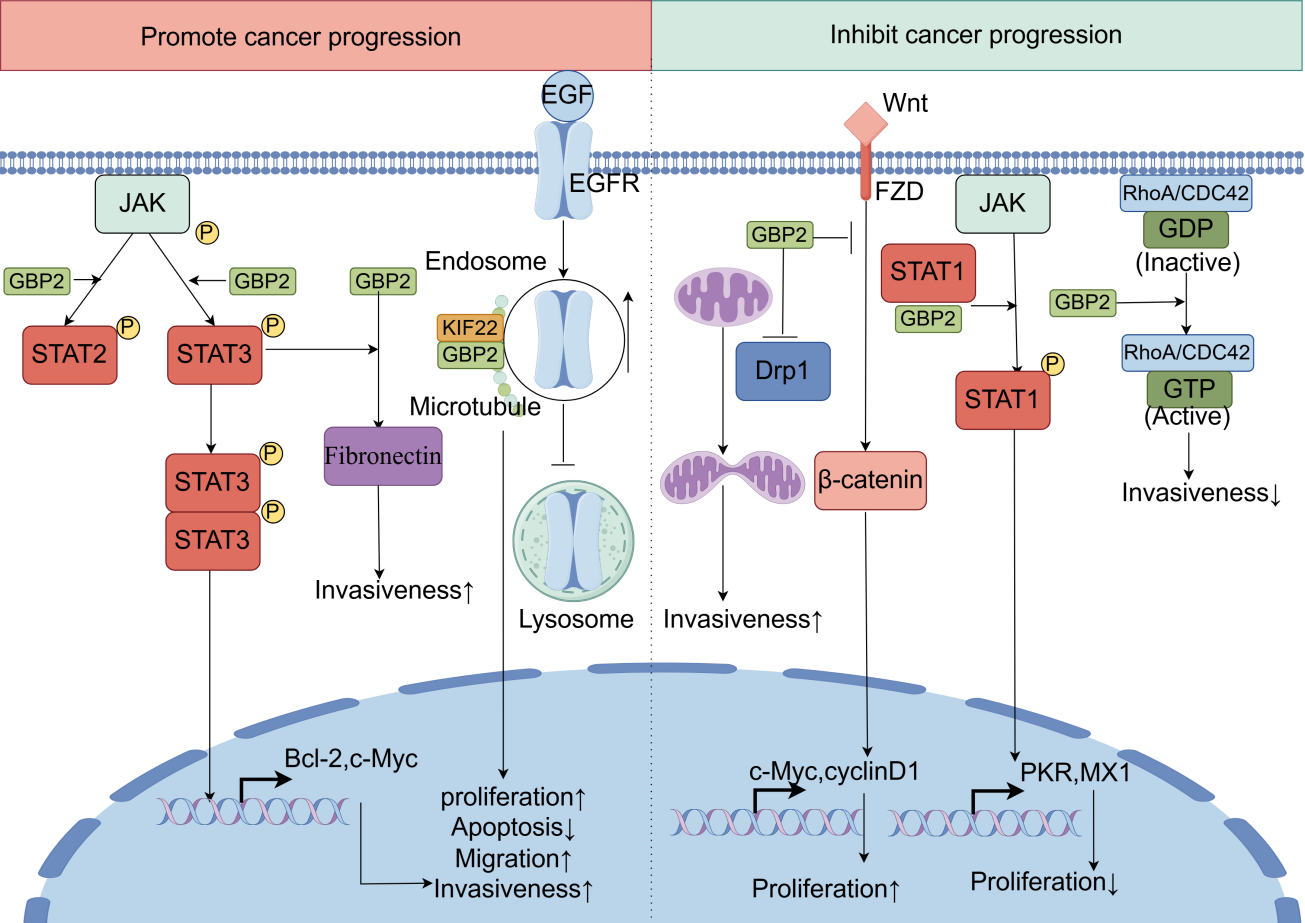
The dual roles of GBP2 in innate immunity and cancer. GBP2 exhibits context-dependent dual roles in tumorigenesis, either promoting or suppressing cancer progression across different cancer types. On one hand, GBP2 acts as a tumor-promoting factor through multiple mechanisms: it significantly upregulates fibronectin expression, which is essential for GBP2-driven cancer cell invasiveness. Additionally, GBP2 enhances the phosphorylation of STAT3 and STAT2, thereby activating the JAK-STAT signaling pathway to facilitate cell migration and invasion. Furthermore, GBP2 directly interacts with KIF22 on microtubules, impeding the trafficking of epidermal growth factor receptor signaling from endosomes to lysosomes for degradation. This leads to sustained epidermal growth factor receptor signaling and promotes tumor progression. On the other hand, GBP2 also demonstrates tumor-suppressive functions: it promotes STAT1 phosphorylation by competing with the phosphatase SH2-containing protein tyrosine phosphatase 1 for STAT1 binding, resulting in reduced tumor proliferative capacity. Moreover, GBP2 inhibits the Wnt/β-catenin signaling pathway, thereby dampening tumor development and metastasis. It also blocks dynamin-related protein 1 translocation from the cytosol to mitochondria, attenuating dynamin-related protein 1-dependent mitochondrial fission and consequently reducing cancer cell invasion. In certain contexts, GBP2 activates CDC42, leading to decreased invasiveness.

GBP2 has emerged as a significant regulator of chemoresistance and a potential therapeutic target in cancer treatment, addressing the major challenge of chemoresistance in achieving effective anticancer outcomes. Recent evidence indicates that GBP2 expression modulates sensitivity to paclitaxel. In triple-negative breast cancer cells, upregulation of GBP2 was found to enhance paclitaxel sensitivity, promote autophagy, and inhibit cell proliferation. This increased drug sensitivity was attenuated upon administration of autophagy inhibitors, suggesting the mechanistic involvement of autophagic processes. Further investigation revealed that GBP2 facilitates autophagy through suppression of the PI3K/AKT/mTOR signaling pathway and via physical interaction with autophagy-related protein 2 (115). Notably, paclitaxel itself can induce GBP2 expression, and GBP2 knockout in chronic myeloid leukemia cells significantly attenuates paclitaxel -induced apoptosis (4). In paclitaxel -resistant colorectal cancer cell lines, both mRNA and protein levels of GBP2 were substantially downregulated compared to their non-resistant counterparts. Ectopic expression of GBP2 in these resistant cells restored paclitaxel sensitivity, resulting in suppressed proliferation, reduced invasion, and increased apoptosis. Mechanistically, GBP2 potentiates the cytotoxic effects of paclitaxel in both sensitive and resistant colorectal cancer models by inhibiting Wnt signaling (11). Beyond paclitaxel-response, GBP2 has also been identified as a potential target of quercetin in melanoma. In murine melanoma B16-F1 cells, quercetin treatment upregulates GBP2 expression, which correlates favorably with prognostic outcomes. Functional studies suggest that GBP2 may restrain melanoma progression by modulating mitochondrial fission and inhibiting invasive behaviors (116). Additionally, GBP2 expression is modulated by endocrine agents: it was significantly upregulated by estradiol and downregulated by tamoxifen in breast cancer models, implicating a role in hormone-therapy response (117). In bladder cancer, intravesical Bacillus Calmette–Guérin immunotherapy has shown efficacy against high-grade non-muscle-invasive disease. GBP2 appears to be specifically involved in the Bacille Calmette–Guérin-responsive gene program, despite exhibiting minimal basal expression in normal urothelium (**Figure 7**) (118).

### 5.5 Context-dependent dual roles of GBP2 in cancer

A substantial body of literature indicates that GBP2 can exhibit both pro-tumorigenic and anti-tumorigenic functions in cancer. The mechanistic basis for this context-dependent duality may be attributed to several factors. First, the proper function of GBP2 relies on precise molecular regulation—including GTP binding, dimerization, and isoprenylation. Point mutations (e.g., S52N) that impair these processes abrogate its activity, suggesting that cell-type-specific differences in its post-translational modifications or protein-interaction networks may lead to divergent phenotypic outcomes. Second, GBP2 may act as a signaling node whose downstream effects are determined by the specific oncogenic networks predominant in a given cellular context. Much like a multifunctional tool, it can be exploited to “construct” pro-tumorigenic programs in some cancers, while in others it contributes to “destructive” anti-tumorigenic mechanisms.

## 6 The role of GBP2 in human diseases beyond infection and cancer

### 6.1 The role of GBP2 in rheumatoid diseases

GBP2 has been implicated in several rheumatoid diseases, including rheumatoid arthritis, lupus erythematosus, lupus nephritis, and primary Sjögren’s syndrome (119–122). In discoid lupus erythematosus, GBP2 ranks among the top ten differentially expressed genes, indicating its potential involvement across multiple stages of the immune response (122). Regarding lupus nephritis, GBP2 expression is significantly upregulated in renal tissues compared to healthy controls. As an integral component of the IFN signaling pathway, GBP2 likely contributes to disease pathogenesis and emerges as a potential biomarker for this condition (120). In primary Sjögren’s syndrome, GBP2 serves as a salivary biomarker that not only distinguishes patients from healthy controls but also differentiates primary Sjögren’s syndrome from systemic lupus erythematosus (121). Furthermore, in rheumatoid arthritis models, GBP2 demonstrates differential expression in both collagen-induced arthritis and collagen-induced arthritis combined with organic dust exposure—particularly within specific lung cell populations—correlating with rheumatoid arthritis progression and associated pulmonary complications (119).

### 6.2 The role of GBP2 in metabolic disorders

GBP2 has been implicated in the pathogenesis of various metabolic disorders, including nonalcoholic fatty liver disease and diabetic complications (16, 123). In the context of nonalcoholic fatty liver disease, GBP2 expression is significantly elevated compared to normal hepatic tissues and contributes to hepatic lipid accumulation through the PPARγ–CD36 axis. Targeting GBP2, along with related signaling molecules such as C-Maf-inducing protein, may thus offer novel therapeutic strategies for the prevention and treatment of this condition (16). Regarding diabetic nephropathy, GBP2 promotes M1 macrophage polarization by activating the Notch1 signaling pathway. Inhibition of GBP2 suppresses Notch1 activation, indicating that GBP2 facilitates canonical Notch signaling—either directly by promoting nuclear translocation of the Notch1 intracellular domain, or indirectly through modulating its acetylation (73). Furthermore, GBP2 exhibits altered expression in the diabetic retina, where it may regulate inflammatory responses via pyroptosis (9, 123). Additionally, GBP2 has been identified as a component of a biomarker panel with potential utility in pharmacotherapeutic research for diabetic retinopathy (124).

### 6.3 The role of GBP2 in neurological disorders

GBP2 has been implicated in the pathogenesis of multiple neurological diseases. In Parkinson’s disease, GBP2—along with C3 and Serping1—is significantly upregulated in the substantia nigra of rats injected with preformed fibrils, identifying it as a marker of reactive astrocytes (125). In Alzheimer’s disease, GBP2 expression in astrocytes is associated with amyloid plaques; it is markedly increased in astrocytes treated with fibrillar amyloid β-protein 42, and immunolabeling in TgF344-AD rat brains shows enhanced GBP2 expression surrounding amyloid plaques compared to wild-type controls (126). Following traumatic brain injury, GBP2 expression is regulated by the JAK2/STAT1 signaling pathway. GBP2 interacts with phosphorylated STAT1, and administration of the JAK2 inhibitor AG490 disrupts this interaction and promotes functional recovery after injury (127). Similarly, after subarachnoid hemorrhage, GBP2 protein levels rise significantly, peaking at 24 hours, and may contribute to pathogenesis by inhibiting the PI3K/AKT pathway (17). In migraine without aura, a rare nonsynonymous mutation in GBP2 (A907G) was identified in patients and absent in controls, suggesting a potential role in vasomotor dysfunction and migraine pathogenesis (128). GBP2 is also included in a five-gene signature predictive of relapse-free survival in multiple sclerosis (129), and has been found to be differentially expressed in human T-lymphotropic virus 1-associated myelopathy/tropical spastic paraparesis (130). Moreover, maternal infection—a known risk factor for schizophrenia—may dysregulate GBP2 through epigenetic mechanisms, potentially linking its antiviral functions to neurodevelopmental pathology (131).

### 6.4 The role of GBP2 in hematological diseases

GBP2 has been implicated in several hematological disorders, where it influences disease progression and demonstrates diagnostic utility. Elevated GBP2 expression is significantly associated with reduced survival in patients with myelodysplastic syndromes, underscoring its prognostic relevance (132). In the context of molecular diagnostics, GBP2 is specifically upregulated in JAK2V617F-positive myelofibrosis. It forms part of a four-gene signature that yields high area under the receiver operating characteristic curve values, effectively distinguishing JAK2V617F^+^ myelofibrosis from other myeloproliferative neoplasms such as polycythemia vera and essential thrombocythemia (90). Furthermore, GBP2 contributes functionally to erythropoiesis. It is downregulated during normal erythroid differentiation and regulates both proliferation and erythroid maturation in TF−1 cells. The microRNA miR−433 negatively modulates hematopoietic proliferation and erythropoiesis by directly targeting GBP2, indicating a post−transcriptional regulatory mechanism in these processes (133).

### 6.5 The role of GBP2 in other diseases

In transplantation medicine, GBP2 contributes to maintaining homeostasis in the peri-implant epithelium, which serves as a critical barrier against inflammatory initiation at implantation sites. Expression levels of GBP2 in the peri-implant epithelium are approximately 8.9-fold higher than in the junctional epithelium, with immunohistochemical analysis confirming moderate staining in peri-implant tissues (134). Additionally, GBP2 has emerged as a promising peripheral blood biomarker for acute cellular rejection. In patients experiencing acute cellular rejection, GBP2 expression is significantly elevated compared to those with hepatitis C or without severe liver dysfunction following transplantation. Using a cut-off value of 20 (GBP2/GAPDH ratio), receiver operating characteristic curve analysis demonstrated 63% sensitivity and 85% specificity for detecting acute cellular rejection. The correlation between GBP2 mRNA levels in peripheral leukocytes and liver grafts, along with its consistent upregulation in allogeneic transplantation models, supports its diagnostic utility—particularly in cases accompanied by severe liver dysfunction (34, 135). In ocular diseases, GBP2 participates in retinal pathophysiology. Its expression is downregulated in both oxygen-induced retinopathy mouse models and hypoxic ARPE-19 cells. Functionally, GBP2 inhibits angiogenesis via the AKT/mTOR/VEGFA signaling axis, suggesting its potential as a therapeutic target for pathological retinal angiogenesis. Knockdown of GBP2 activated the AKT/mTOR pathway *in vitro*, whereas overexpression produced inhibitory effects (7). Under chronic hypoxic conditions, GBP2 levels were elevated in the vitreous and accompanied by increased retinal mRNA expression, indicating its potential as an early marker of photoreceptor response to hypoxia (136). Furthermore, GBP2 has been identified as a key regulator in coronary artery disease, where it orchestrates relevant biological processes (137). M The protein also appears to play a significant role in acute respiratory distress syndrome, showing promise as both a diagnostic indicator and therapeutic target (138). The regulatory role of GBP2 in non-infectious human diseases are summarized in **Table 2**.

**Table 2:** Summary of GBP2 dysregulation, mechanisms, and functional roles in non-infectious human diseases.

Disease	GBP2 expression change	Mechanism	Main function	References
Cancer				
Glioblastoma	↑	Promotes fibronectin induction via STAT3 pathway	Promotes tumor cell migration and invasion	(13)
Clear Cell Renal Cell Carcinoma	↑	Enhances phosphorylation of STAT2/STAT3, triggering JAK-STAT signaling	Promotes cell migration and invasion; Associated with immune infiltration and poor prognosis	(14, 96)
Glioma	↑	Interacts with KIF22 to regulate KIF22/epidermal growth factor receptor signaling	Promotes cell proliferation and migration	(97)
Esophageal Squamous Cell Carcinoma	↑	Unclear	Associated with proliferative and neoplastic phenotype	(35)
Cutaneous Melanoma	↓	Inhibits Wnt/β-catenin signaling	Suppresses metastasis; Acts as a potential tumor suppressor	(6)
Breast Cancer (TNBC, paclitaxel -Resistance)	↓	Inhibits PI3K/AKT/mTOR pathway; Interacts with autophagy-related protein 2	Increases paclitaxel sensitivity; Enhances autophagy; Suppresses cell growth	(115)
Colorectal Cancer (paclitaxel -Resistant)	↓	Suppresses Wnt signaling	Increases paclitaxel sensitivity; Decreases proliferation and invasion; Increases apoptosis	(11)
Osteosarcoma	↓	Unclear	Suppresses cell migration and invasion	(100)
Rheumatological Diseases				
Lupus Nephritis	↑	Unclear; Part of interferon signaling pathway	Potential contributor to pathogenesis; Potential biomarker	(120)
Primary Sjögren’s Syndrome	↑(Saliva)	Unclear	Salivary biomarker for diagnosis and differentiation from systemic lupus erythematosus	(121)
Discoid Lupus Erythematosus	Unclear	Unclear	Involved in immune response stages	(122)
Rheumatoid Arthritis (with lung complication)	↑	Unclear	Correlates with disease and lung complications	(119)
Metabolic Disorders				
Non-alcoholic Fatty Liver Disease	↑	Acts via the PPARγ–CD36 axis	Promotes hepatic lipid accumulation	(16)
Diabetic Nephropathy	↑	Activates Notch1 signaling pathway	Promotes M1 macrophage polarization	(73)
Diabetic Retinopathy	↑	Unclear; May regulate inflammation through pyroptosis	Potential component of a biomarker panel	(9, 123, 124)
Neurological Disorders				
Parkinson’s Disease	↑	Unclear	Marker of reactive astrocytes	(125)
Alzheimer’s Disease	↑ (Astrocytes)	Unclear	Associated with amyloid plaques	(126)
Traumatic Brain Injury	↑	Interacts with p-STAT1 (JAK2/STAT1 pathway)	Contributes to pathogenesis; Inhibition promotes recovery	(127)
Subarachnoid Hemorrhage	↑	Inhibits PI3K/AKT pathway	Contributes to pathogenesis	(17)
Migraine without Aura	Mutation (A907G)	Unclear	Potential role in vasomotor dysfunction and pathogenesis	(128)
Multiple Sclerosis(relapse-free survival)	↓	Unclear	Predictive biomarker for relapse-free survival	(129)
Hematological Diseases				
Myelodysplastic Syndromes	↑	Unclear	Prognostic marker (associated with shorter survival)	(132)
JAK2V617F^+^ Myelofibrosis	↑	Unclear	Diagnostic biomarker (part of a gene signature)	(90)
Erythropoiesis (TF-1 cells)	↓	Regulated by miR-433	Regulates proliferation and erythroid maturation	(133)
Acute Myeloid Leukemia	↓	Regulation of PI3K/AKT pathway	Suppresses cell proliferation and reduces apoptosis	(106)
Transplantation				
Acute Cellular Rejection	↑(Blood)	Unclear	Potential peripheral blood biomarker for diagnosis	(34, 135)
Peri-implant Epithelium	↑	Unclear	Maintains homeostasis	(134)
Ocular Diseases				
Pathological Retinal Angiogenesis	↓	Inhibits AKT/mTOR/VEGFA signaling	Inhibits pathological retinal angiogenesis	(7)
Chronic Retinal Hypoxia	↑(Vitreous)	Unclear	Potential early marker of photoreceptor response to hypoxia	(136)
Cardiovascular & Respiratory				
Coronary Artery Disease	↑	Unclear	Orchestrates relevant biological processes	(137)
Acute Respiratory Distress Syndrome	↑	Unclear	Potential diagnostic and therapeutic target	(138)

## 7 Therapeutic implications of GBP2: diagnostic biomarker, differential diagnostic utility, and therapeutic target

Accumulating evidence underscores the significant potential of GBP2 as a diagnostic biomarker, a tool for differential diagnosis, and a promising therapeutic target across various human diseases. In tuberculosis, GBP2 is consistently downregulated and has been identified as a hub gene with considerable diagnostic value; its significantly reduced expression in tuberculosis patients supports its utility for treatment monitoring (56). Additionally, during dengue infection, plasma GBP2 levels emerge as a potential biomarker for disease severity, correlating with key clinical manifestations (42). Beyond infectious diseases, GBP2 exhibits distinctive expression patterns that aid in differential diagnosis. For example, it serves as a salivary biomarker that not only identifies patients with primary Sjögren’s syndrome but also differentiates this condition from systemic lupus erythematosus (121).

Therapeutically, GBP2 plays a context-dependent role in modulating cancer treatment response. It enhances sensitivity to anti-PD-1 immunotherapy and suppresses tumor growth in colorectal cancer (76). In triple-negative breast cancer, GBP2 increases paclitaxel sensitivity by promoting autophagy through suppression of the PI3K/AKT/mTOR pathway and physical interaction with autophagy-related protein 2 (115). Similarly, in paclitaxel -resistant colorectal cancer models, restoring GBP2 expression re-sensitizes cells to paclitaxel via inhibition of Wnt signaling, leading to suppressed proliferation and invasion, along with increased apoptosis (11). Collectively, these findings position GBP2 as a multifaceted player in clinical medicine—functioning as a diagnostic indicator, a discriminator among complex diseases, and a modulator of treatment response—with mechanistic involvement across JAK-STAT, Wnt, and PI3K/AKT/mTOR signaling pathways.

## 8 Limitations of current evidence and knowledge gaps

While this review synthesizes the expanding roles of GBP2 across diseases, a critical appraisal reveals significant limitations in the current evidence base and highlights crucial knowledge gaps that future research must address. A primary concern is the heavy reliance on *in vitro* models and preclinical studies, which form the bulk of the mechanistic evidence (13, 49, 97). While these models are invaluable for hypothesis generation, they often lack the complexity of the human tumor microenvironment or tissue-specific physiology, raising questions about the translational relevance of these findings. The scarcity of genetically engineered mouse models that specifically manipulate GBP2 expression in a spatiotemporal manner limits our understanding of its systemic and cell-autonomous functions *in vivo*. Additionally, substantial methodological limitations also exist. Many studies utilize bulk transcriptomic data to correlate GBP2 expression with clinical outcomes (83, 84). This approach fails to account for cellular heterogeneity within tumors. The seemingly contradictory association of high GBP2 with both favorable immune activation and immunosuppressive checkpoint expression could potentially be resolved by single-cell analyses to determine which specific cell types (e.g., tumor cells, T cells, macrophages) express GBP2 in different contexts (76, 96). Furthermore, species differences between human and murine GBP systems are often overlooked. For example, the human GBP family has seven members, while mice have eleven, leading to potential functional redundancy or divergence that complicates the extrapolation of findings from mouse models to human diseases (1).

## 9 Conclusion and perspectives

Recent advances have unveiled the multifaceted roles of GBP2 in human diseases—including cancer, neurological, metabolic, and autoimmune disorders—highlighting its promising potential as a therapeutic target. Functioning both within and beyond the guanylate-binding protein family, GBP2 mediates cell-autonomous innate immunity against viral, bacterial, and parasitic infections through mechanisms such as furin inhibition, inflammasome activation, and targeted protein trafficking. In cancer, it exhibits context−dependent duality, modulating key pathways—including JAK−STAT, Wnt/β−catenin, and immune signaling—to influence tumor progression, metastasis, and therapy response.

To resolve the paradox of its opposing functions, we propose a unifying hypothesis in which GBP2’s biological output is shaped by the molecular milieu—including cell−type−specific interaction partners and upstream signaling cues—together with its post−translational modification status. In growth factor−enriched, pro−tumorigenic niches (e.g., under platelet-derived growth factor stimulation), GBP2 is co−opted to potentiate oncogenic pathways such as JAK−STAT, thereby fostering invasive behavior. Conversely, in homeostatic or defense−primed contexts, GBP2 exerts tumor−suppressive effects by harnessing its GTPase activity and membrane localization properties to disrupt core motility machinery, including cytoskeletal remodeling and mitochondrial fission.

Future research on GBP2 should prioritize elucidating the molecular determinants underlying its context−dependent roles in cancer and other diseases. Key areas include delineating how cell−type−specific interactors, signaling environments, and post−translational modifications—such as GTP binding, dimerization, and isoprenylation—dictate its functional outcomes. In−depth mechanistic studies are also warranted to explore GBP2’s regulation of critical pathways, including JAK−STAT, Wnt/β−catenin, PI3K/AKT/mTOR, and Rho GTPase cascades across different pathological contexts. Substantial knowledge gaps remain in understanding GBP2’s immunomodulatory roles. While its contributions to inflammasome activation and T−cell polarization have been established, the detailed molecular cascades and functional consequences for the overall immune landscape in tumors or autoimmune diseases are not fully delineated. Finally, the development of GBP2−targeted agents—such as small−molecule modulators and biologicals—represents a promising translational direction. Efforts to validate GBP2 as a diagnostic or prognostic biomarker in infections, cancers, and autoimmune diseases should also be strengthened through multi−center clinical studies.

In summary, the role of GBP2 in human diseases appears to have been significantly underestimated, underscoring the need for expanded investigation into its molecular functions and therapeutic applicability.

## Glossary

GBPs: Guanylate-binding proteins
GBP2: Guanylate-Binding Protein 2
PI3K: Phosphatidylinositol 3-kinase
AKT: Ak strain transforming
mTOR: Mammalian target of rapamycin
Wnt: Wingless-type MMTV integration site family
JAK: Janus kinase
LG: Large GTPase domain
MD: Middle domain
GED: GTPase effector domain
IFN: Interferon
GTP: Guanosine triphosphate
GDP: Guanosine diphosphate
GMP: Guanosine monophosphate
STAT1: Signal transducer and transcription activator 1
IRF: Interferon regulatory factor
NF-κB: Nuclear factor κb
LPS: Lipopolysaccharide
NLRP3: NOD-like receptor protein 3
T3SS: Type III secretion systems
TNF-α: Tumor necrosis factor-α
Notch1: Neurogenic locus notch homolog protein 1
AIM2: Absent in melanoma 2
KIF22: Kinesin family member 22
BAK: BCL-2 antagonist/killer 1
PD-1: Programmed cell death 1
PD‐L1: Programmed cell death ligand 1
